# Oncocytic Papillary Cystadenoma in the Tubarial Gland: A Case Report and Literature Review

**DOI:** 10.7759/cureus.117181

**Published:** 2026-09-29

**Authors:** Reuben P Powell, Celeste Wagner, Tyler A Janz, Viran J Ranasinghe, Cecilia Clement

**Affiliations:** 1 Pathology, University of Texas Medical Branch, Galveston, USA; 2 Otolaryngology, University of Texas Medical Branch, Galveston, USA; 3 Otolaryngology - Head and Neck Surgery, University of Texas Medical Branch, Galveston, USA

**Keywords:** head and neck neoplasms, major salivary glands, minor salivary glands, nasopharyngeal salivary neoplasms, oncocytic papillary cystadenoma, tubarial glands

## Abstract

Oncocytic papillary cystadenoma is a rare, benign lesion primarily affecting the major and minor salivary glands of the head and neck. Occurrence in the nasopharynx is exceedingly rare. Herein, we describe an oncocytic papillary cystadenoma on a tubarial gland of the nasopharynx. The tubarial glands are conglomerates of predominantly mucous glands overlying the torus tubarius. These glands were identified incidentally on ⁶⁸Ga-PSMA-11 positron emission tomography/computed tomography (PET/CT) performed for staging in prostate cancer patients, owing to their expression of prostate-specific membrane antigen (PSMA), a characteristic shared with the major salivary glands. This led to increased scientific discussions that have increased our understanding of the anatomy, histology, and physiologic role of these glands, as well as their implications in disease and therapy.

A 77-year-old male smoker presented with a six-month history of imbalance and bilateral ear fullness. Subsequent endoscopy revealed an erythematous, exophytic mass on the left torus tubarius. The mass was removed by excisional biopsy and diagnosed as an oncocytic papillary cystadenoma.

Oncocytic papillary cystadenoma of the nasopharyngeal tubarial glands is uncommon. Based on recent investigations and developments, our understanding of this anatomic structure has improved. Continued investigation and awareness of this concealed salivary gland is needed to further improve our understanding of its function and pathology.

## Introduction

Cystadenoma is a rare salivary gland tumor, comprising approximately 1%-4% of benign salivary gland tumors, of which oncocytic papillary cystadenoma (OPC) is a subset. The tumor occurs in both major and minor salivary glands, with reported site distributions varying widely across series. OPC is a benign lesion that is classified by the World Health Organization as a neoplasm; however, a genetic driver has not been identified [1]. In addition, oncocytic metaplasia may be associated with a smoking history and age over 60 years [2,3]. Whether OPC may represent ductal hyperplasia/metaplasia rather than true neoplasia is not fully resolved. There are few cases in the literature of OPC occurring in the nasopharynx [4].

The tubarial glands (TG) are a pair of predominantly mucous glands overlying the torus tubarius, the cartilaginous protrusion of the eustachian tube [5]. These structures have been highlighted recently because of their prostate-specific membrane antigen (PSMA) positivity, a characteristic shared with the major salivary glands. This has led to investigations into their clinical significance, both as a potential organ at risk during radiotherapy and PSMA-targeted radionuclide therapy, as well as a site of potentially under-recognized pathology [6].

## Case presentation

A 77-year-old male patient presented with a six-month history of bilateral ear fullness and imbalance. Subsequent endoscopy identified an erythematous, exophytic mass on the left torus tubarius (Figure 1). Computed tomography was performed but did not identify a discrete mass. The patient underwent endoscopic excisional biopsy for definitive diagnosis.

**Figure 1:**
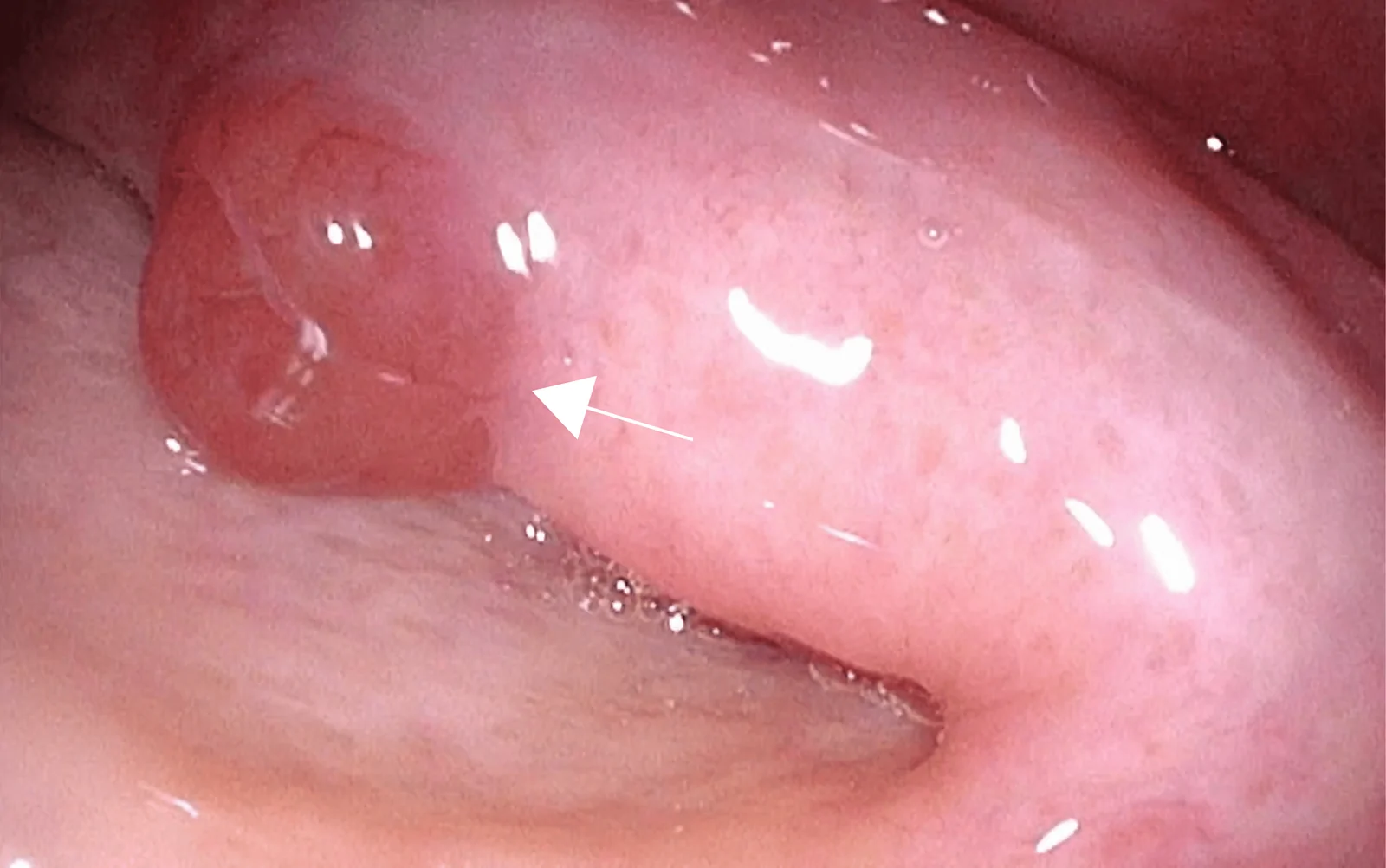
Endoscopic view of the posterior nasopharynx with abnormal and irregular mucosa (arrow) along the left torus tubarius.

Grossly, the specimen was a tan-pink polypoid soft tissue fragment (0.5x0.3x0.2 cm). Histological examination revealed a well-circumscribed, unencapsulated lesion consisting of cysts filled with eosinophilic fluid and papillary projections (Figure 2A). Simple papillary projections into the lumen of the cystic spaces were noted (Figure 2B). The cysts were separated by fibrous connective tissue without inflammation and were lined by single and bilayer oncocytic cells (Figure 2C). Cytologically, the oncocytic cells presented abundant eosinophilic granular cytoplasm, well-defined cell borders, bland nuclei and occasional prominent nucleoli. No invasion or atypia was identified. The morphologic findings were consistent with a diagnosis of oncocytic papillary cystadenoma.

**Figure 2:**
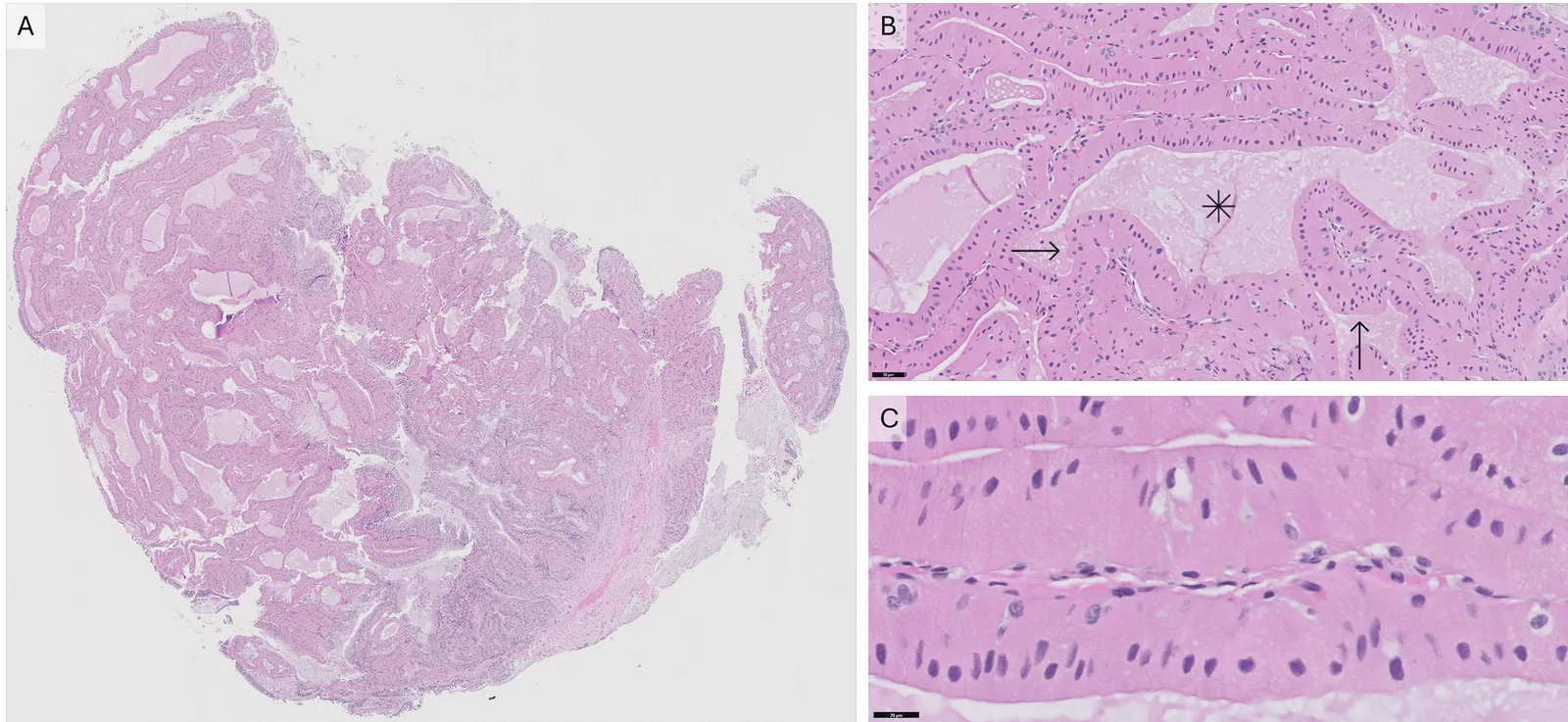
Histology of tubarial gland lesion. Low magnification revealing a well-circumscribed, unencapsulated multilocular cystic lesion (A). Cystic spaces (asterisk) with simple papillary projections (arrows) into the cysts lumen lined by a single and bilayer oncocytic epithelial cells (B). High magnification of bilayer oncocytic epithelium (C).

No additional imaging or vestibular testing was pursued, as the bilateral symptoms were considered clinically unrelated to the small unilateral tubarial lesion. Given the benign nature of the lesion, no further treatment was pursued, and the patient will be followed up as needed. A year later, the patient was seen at the otolaryngology clinic for a follow-up of his long-standing hearing loss, and there were no other issues related to his lesion.

## Discussion

The purpose of this case report is to illustrate an uncommon entity occurring in a rare location, as well as continue to highlight the growing knowledge and understanding of the tubarial glands.

Cystadenoma of the salivary glands is a rare benign tumor usually presenting as a slow-growing mass. Most cases occur in the major and minor salivary glands of the oral cavity, specifically the parotid, lip, buccal, or palatal salivary glands and the mean age of occurrence is in the fifth to seventh decade of life [1,7]. Cystadenoma can be subdivided into mucinous and papillary types. The latter has well-defined unilocular or multilocular cysts, with intraluminal papillary projections. The lining epithelial cells are cuboidal or columnar, and usually two layers in thickness. Oncocytic change is a frequent feature of cystadenoma and can be extensive, as in our patient [8]. Oncocytic papillary cystadenoma is often confused with Warthin tumor because of its histologic similarity: papillary structures, oncocytic cells with rich eosinophilic cytoplasm, and prominent nucleoli. However, Warthin tumors can be distinguished by their lymphoid-rich stroma. Another differential diagnosis is oncocytoma, which is characterized by polyhedral oncocytic cells. Cystic change can be seen in this tumor, but the papillary pattern is not typical of oncocytoma [9].

The tubarial glands (TG) are an aggregate of predominantly mucous glandular tissue in the nasopharynx overlying the torus tubarius and extending to posterolateral pharyngeal recess (Fossa of Rosenmüller) [10]. Embryologically, the primordial salivary glands of the nasopharynx begin to appear in the 11th week of life just below the tubal orifice, where undifferentiated precursor cells invaginate into the lamina propria to form cylinders. From this epicentre, salivary gland development spreads anterior-inferiorly toward the uvula and pharynx, posteriorly to the Fossa of Rosenmüller, and into the eustachian tube. By the 14th week, the spread progresses superiorly and anteriorly to the pharyngeal roof and nose [11]. At 23 weeks, the salivary glands are nearly fully formed. In an adult, the TG are approximately 4 cm [12]. Histologically, the surface epithelium of the nasopharynx can be a mix of ciliated respiratory and stratified squamous mucosa; in up to 41% of Southeast Asians, the mucosa can contain melanin pigment [13]. Various studies have shown that the TGs are composed of mucous acinar cells with serous demilunes, which are connected to the epithelium by excretory and squamous ducts [14,15]. The TGs are macroscopic structures, which were identified using radio-labeled PSMA and subsequently gained notoriety as a potentially under-investigated anatomic structure [6].

Some scholars have pointed out key features that may indicate a classification as a non-organ component of the respiratory tract, namely anatomic location, lack of encapsulation, and overlying epithelium, as well as physiological similarities to respiratory glands [16]. Other scholars have undertaken histological and immunohistochemical studies that have highlighted the salivary gland-like nature of the tubarial glands, with closest resemblance to the palatal (minor) salivary glands [15]. This is not a new topic of discussion, as the early naming of the anatomic region was debated as to whether the term pharynx, implying digestive tract, applied; yet, in the same article, it is illustrated how the torus tubarii are uniquely situated with a mixed ciliated columnar (respiratory) and stratified squamous (oral) epithelium [13]. Perhaps, it is less beneficial to ask which anatomic system it most specifically resembles than to investigate how this unique structure contributes to both oral and respiratory physiology and pathology. Indeed, some have suggested that salivary glands be considered on a spectrum rather than as discrete categories [17].

Oncocytic papillary cystadenoma is not listed in the nasopharyngeal section of the WHO classification; rather, like other salivary gland neoplasms that are not unique to a single site, it is classified within the salivary gland tumors chapter and can arise wherever salivary or seromucinous glandular tissue is present, including the nasopharynx. Its occurrence on the tubarial glands is therefore expected on histogenetic grounds. A related lesion, melanotic oncocytic metaplasia of the nasopharynx is a rare entity that appears to occur predominantly in East and Southeast Asian male smokers and has a propensity to occur on the tubarial glands [18,19]. More studies are needed to better understand the role of the tubarial glands, which are believed to play a significant part in nasopharyngeal lubrication. Damage to these glands can exacerbate xerostomia and dysphagia, both from external-beam radiotherapy for head and neck cancer and from PSMA-targeted radionuclide therapy for prostate cancer [6,20].

## Conclusions

Oncocytic papillary cystadenoma of the tubarial gland represents a rare and intriguing phenomenon, and tumors originating from this region may be underreported. The recent discovery of this gland has sparked renewed scientific interest, presenting an exciting opportunity for further exploration in both research and clinical practice. Our case adds to awareness of pathology arising from this region that can potentially be missed on imaging. It is worth emphasizing that Valstar et al. did not claim to have discovered a new organ; they proposed the name "tubarial glands" for previously overlooked macroscopic salivary gland locations and framed them as a *potential new organ at risk for radiotherapy*. Glandular tissue in this location was, moreover, described well before the PSMA work, and whether these glands constitute a discrete named entity remains debated. Improved understanding of this region holds potential to advance our understanding of a range of head and neck diseases and their management.
